# ﻿Leaving no stone unturned: three additional new species of *Atractus* ground snakes (Serpentes, Colubridae) from Ecuador discovered using a biogeographical approach

**DOI:** 10.3897/zookeys.1121.89539

**Published:** 2022-09-15

**Authors:** Alejandro Arteaga, Amanda Quezada, Jose Vieira, Juan M. Guayasamin

**Affiliations:** 1 Biodiversity Field Lab (BioFL), Khamai Foundation, Quito, Ecuador Biodiversity Field Lab (BioFL), Khamai Foundation Quito Ecuador; 2 ExSitu, Quito, Ecuador ExSitu Quito Ecuador; 3 Laboratorio de Biología Evolutiva, Instituto Biósfera, Colegio de Ciencias Biológicas y Ambientales COCIBA, Universidad San Francisco de Quito USFQ, Quito, Ecuador Universidad San Francisco de Quito USFQ Quito Ecuador

**Keywords:** Biodiversity, biogeography, Colubridae, fossorial, phylogeny, new species, taxonomy

## Abstract

The genus *Atractus* includes 146 species of cryptozoic snakes occurring from Panama to northeastern Argentina. Here, a molecular phylogeny of this genus is presented, which encompasses 29% (= 42; six are included here for the first time) of the species currently recognized. Morphological and phylogenetic support is found for three new species of ground snakes, which are described here based on their unique combination of molecular, meristic, and color pattern characteristics. The name *A.arangoi* Prado, 1939 is revalidated for a Colombian snake species previously subsumed under *A.major* Boulenger, 1894 based on new material collected in Ecuador. Reidentifications are provided for *Atractus* voucher specimens and sequences deposited in GenBank. With these changes, the number of *Atractus* reported in Ecuador increases from 27 to 31 species. Finally, attention is given to the importance of using a biogeographical framework that includes molecular data and a comprehensive geographic sampling when proposing species limits in complex taxonomic groups.

## ﻿Introduction

*Atractus* Wagler, 1828 is the most speciose snake genus in the world (Uetz et al. 2022). There are 146 known species, and these numbers are likely to rise with the exploration of remote mountain ranges, the use of molecular tools in *Atractus* systematics, and the application of a biogeographical framework when establishing limits between species.

In Ecuador, the exploration of remote mountain ranges (e.g., the Cordillera de Guacamayos, Sumaco Volcano, and the Cordillera del Cóndor) within the last two decades has resulted in the discovery of at least six species of *Atractus*, including the most heavy-bodied and strikingly colored in the genus (Myers and Schargel 2006; Schargel et al. 2013; Arteaga et al. 2017; Passos et al. 2018; Melo-Sampaio et al. 2021). Unlike other snake genera inhabiting the same mountain ranges (e.g., *Dipsas*; see Arteaga et al. 2018), snakes in the genus *Atractus* inhabiting remote cloud forests and inter-Andean valleys are generally considered rare. Some are known only from their type localities (e.g., *A.cerberus*Arteaga et al., 2017) whereas for some species the males (e.g., *A.atlas*Passos et al., 2018) or juveniles (e.g., *A.touzeti*Schargel et al., 2013) have not yet been reported. All of this suggests that *Atractus* in general, with the exception of some locally abundant species (e.g., *A.marthae* Meneses-Pelayo & Passos, 2019), are difficult to find. Thus, species inhabiting poorly visited areas may remain undetected without long-term projects focused on cryptozoic herpetofauna (Myers 2003).

The use of molecular tools in *Atractus* systematics is also likely to increase the rate at which new species in this genus are detected and described. Only seven species of *Atractus* have been described using molecular data in addition to meristic and color pattern characteristics (Arteaga et al. 2017; Melo-Sampaio et al. 2019; Melo-Sampaio et al. 2021). Some of these new species were previously considered to be widespread, polychromatic, and difficult to diagnose (Savage 1960). Therefore, they probably would have never been detected using meristics and other morphological data alone. Furthermore, only approximately 30% of the current known diversity of the genus has been included in published phylogenetic analyses (i.e., Arteaga et al. 2017; Passos et al. 2022), and even a smaller percentage of the included species have been thoroughly sampled throughout their range. This lack of information presents both a challenge and an opportunity to uncover further cryptic diversity within the genus.

Finally, a mention should be made about the importance of using a biogeographical framework that includes molecular data and species distribution models (when the number and quality of locality records is sufficient for these analyses; see van Proosdij et al. 2015) when defining species limits within *Atractus*. Finding ground snakes along the Andes has showed us (Arteaga et al. 2013, 2017) and other authors (Savage 1955, 1960; Cisneros-Heredia 2005; Salazar-Valenzuela et al. 2014) that snakes in this genus have lower dispersal capacity than other colubrids and many species are endemic to a single mountain range or restricted to an isolated inter-Andean valley. Thus, the presence of the same *Atractus* species in two geographically isolated areas that are climatically and floristically distinct and are separated from each other by tens or even hundreds of kilometers of discontinuous habitat is unlikely. An example of this scenario is *A.gigas* Myers & Schargel, 2006, a species previously considered to be endemic to the Pacific slopes of the Andes in Ecuador (Myers and Schargel 2006; Tolhurst et al. 2010; Arteaga et al. 2013), but later reported on the Amazonian slopes of the Andes in Peru (Passos et al. 2010). Although specimens from both localities may resemble each other in lepidosis, they differ in coloration, ecological requirements, and phylogenetic affinities. More recently, without explanation, but probably based on similarities in meristics, Passos et al. (2022) proposed the reidentification of 15 specimens of *Atractus* having sequences deposited in GenBank. Given that some of these reidentifications involve type series and the majority of them were done without providing an explanation, their validity is evaluated in this work.

To help clear the waters of *Atractus* taxonomy, in this work we present a curated phylogeny of the genus, reidentify *Atractus* sequences in GenBank, present the description of three new species, and provide the revalidation of a taxon previously subsumed under *A.major*.

## ﻿Materials and methods

### ﻿Ethics statement

This study was carried out in strict accordance with the guidelines for use of live amphibians and reptiles in field research (Beaupre et al. 2004) compiled by the American Society of Ichthyologists and Herpetologists (**ASIH**), the Herpetologists’ League (**HL**) and the Society for the Study of Amphibians and Reptiles (**SSAR**). All procedures with animals (see below) were reviewed by the Ministerio del Ambiente, Agua y Transición Ecológica (**MAATE**) and specifically approved as part of obtaining the following field permits for research and collection: MAE-DNB-CM-2015-0017 (granted to Universidad Tecnológica Indoamérica), MAE-DNB-CM-2018-0105 and MAATE-DBI-CM-2022-0245 (granted to Universidad San Francisco de Quito), and 004-AIC-DPC-B-MAE-18 (granted to Universidad del Azuay). Specimens were euthanized with 20% benzocaine, fixed in 10% formalin or 90% ethanol, and stored in 70% ethanol. Museum vouchers were deposited at Museo de Zoología de la Universidad Tecnológica Indoamérica (**MZUTI**), Museo de Zoología de la Universidad San Francisco de Quito (**ZSFQ**), Museo de Zoología de la Universidad del Azuay (**MZUA**), and the herpetology collection at Bioparque Amaru (**AMARU**). Specimens labeled JMG were also deposited at ZSFQ.

### ﻿Common names

Criteria for common name designation are as proposed by Caramaschi et al. (2006) and Coloma and Guayasamin (2011–2017), reviewed by Arteaga et al. (2019). These are as follows (in order of importance): (i) the etymological intention (implicit or explicit) that the authors used when naming the species (specific epithet); (ii) a common name that is already widely used in the scientific literature; (iii) a common name that has an important ancestral or cultural meaning; (iv) a common name based on any distinctive aspect of the species (distribution, morphology, behavior, etc.).

### ﻿Morphological data

Our terminology for *Atractus* cephalic shields follows Savage (1960), diagnoses and descriptions generally follow Passos et al. (2009a), and ventral and subcaudal counts follow Dowling (1951). We examined comparative alcohol-preserved specimens from the herpetology collections at MZUTI, MZUA, ZSFQ, American Museum of Natural History (**AMNH**), Museo de Zoología de la Pontificia Universidad Católica del Ecuador (**QCAZ**), and Muséum National d’Histoire Naturelle (**MNHN**) (Table 1). Morphological measurements were taken with measuring tapes to the nearest 1 mm, or with digital calipers to the nearest 0.1 mm. Abbreviations are as follows: snout-vent length (**SVL**); tail length (**TL**). Sex was determined by establishing the presence/absence of hemipenes through a subcaudal incision at the base of the tail unless hemipenes were everted.

**Table 1.** Locality data for specimens examined in this study. Coordinates represent actual GPS readings taken at the locality of collection or georeferencing attempts from gazetteers under standard guidelines, although some variation from the exact collecting locality will be present. Similarly, elevations are taken from Google Earth and may not exactly match the elevations as originally reported.

Species	Voucher	Country	Province	Locality	Latitude, Longitude	Elev. (m)
*A.arangoi*	**DHMECN 8343**	Ecuador	Sucumbíos	Bloque 27	0.32271, -76.19300	264
*A.arangoi*	**ZSFQ 4947**	Ecuador	Napo	Jatun Sacha Biological Station	-1.06633, -77.61640	423
*A.arangoi*	**ZSFQ 4948**	Ecuador	Napo	Jatun Sacha Biological Station	-1.06633, -77.61640	423
*A.discovery* sp. nov.	**MZUA.RE.0466**	Ecuador	Morona Santiago	Campamento Arenales	-2.59253, -78.56507	2057
*A.discovery* sp. nov.	**ZSFQ 4936**	Ecuador	Azuay	Amaluza	-2.61583, -78.56538	2002
*A.discovery* sp. nov.	**ZSFQ 4937**	Ecuador	Azuay	Amaluza	-2.61583, -78.56538	2002
*A.major*	**MNHN 0.6149**	Ecuador	–	–	–	–
*A.major*	**QCAZ 11565**	Ecuador	Orellana	Tambococha	-0.97839, -75.42569	194
*A.major*	**QCAZ 11587**	Ecuador	Orellana	Tambococha	-1.03981, -75.44849	210
*A.major*	**QCAZ 11596**	Ecuador	Orellana	Tambococha	-0.97839, -75.42569	194
*A.major*	**QCAZ 11809**	Ecuador	Pastaza	Campo Villano B	-1.45745, -77.44455	331
*A.major*	**QCAZ 4691**	Ecuador	Pastaza	Río Sarayakillo	-1.72754, -77.48048	434
*A.major*	**QCAZ 4895**	Ecuador	Orellana	Vía Pompeya Sur-Iro	-0.99307, -76.24904	246
*A.major*	**QCAZ 7881**	Ecuador	Sucumbíos	Pañacocha	-0.44791, -76.07097	240
*A.major*	**QCAZ 7896**	Ecuador	Orellana	Vía Pompeya Sur-Iro	-0.99320, -76.24907	246
*A.major*	**QCAZ 8040**	Ecuador	Napo	Comunidad Gareno	-1.04856, -77.37742	334
*A.major*	**QCAZR 11744**	Ecuador	Pastaza	Lorocachi	-1.65567, -75.96886	212
*A.major*	**ZSFQ 4955**	Ecuador	Morona Santiago	Macas-Riobamba	-2.25674, -78.16797	1148
*A.michaelsabini* sp. nov.	**AMNH 18325**	Ecuador	El Oro	El Chiral	-3.63825, -79.59723	1841
*A.michaelsabini* sp. nov.	**AMNH 22110**	Ecuador	El Oro	La Chonta	-3.56585, -79.85144	1025
*A.michaelsabini* sp. nov.	**AMNH 22111**	Ecuador	El Oro	La Chonta	-3.56585, -79.85144	1025
*A.michaelsabini* sp. nov.	**DHMECN 7644**	Ecuador	Azuay	Reserva Yunguilla	-3.22684, -79.27520	1748
*A.michaelsabini* sp. nov.	**DHMECN 7645**	Ecuador	Azuay	Reserva Yunguilla	-3.22684, -79.27520	1748
*A.michaelsabini* sp. nov.	**QCAZ 7887**	Ecuador	El Oro	Guanazán	-3.44139, -79.49417	2596
*A.michaelsabini* sp. nov.	**QCAZ 7902**	Ecuador	El Oro	Guanazán	-3.44668, -79.49051	2663
*A.michaelsabini* sp. nov.	**QCAZ 9643**	Ecuador	El Oro	El Panecillo	-3.46753, -79.48248	2775
*A.michaelsabini* sp. nov.	**QCAZ 9652**	Ecuador	El Oro	El Panecillo	-3.46753, -79.48248	2775
*A.michaelsabini* sp. nov.	**ZSFQ 4938**	Ecuador	Azuay	Corraleja	-3.38740, -79.22785	2660
*A.michaelsabini* sp. nov.	**ZSFQ 4939**	Ecuador	El Oro	Guanazán	-3.46753, -79.48248	2750
*A.pachacamac*	**ZSFQ 4954**	Ecuador	Morona Santiago	Macas-Riobamba	-2.24087, -78.27632	1644
*A.resplendens*	**ZSFQ 4953**	Ecuador	Tungurahua	Montañas de San Antonio	-1.43413, -78.40726	2655
*A.resplendens*	**ZSFQ 4952**	Ecuador	Tungurahua	Montañas de San Antonio	-1.43413, -78.40726	2655
*A.resplendens*	**ZSFQ 4951**	Ecuador	Tungurahua	Montañas de San Antonio	-1.43413, -78.40726	2655
*A.roulei*	**MNHN 1906.0243**	Ecuador	Chimborazo	Alausí	-2.20636, -78.84611	2400
*A.roulei*	**MZUA.RE.0080**	Ecuador	Azuay	Miguir, 10 km E of	-2.78771, -79.37132	2596
*A.roulei*	**MZUTI 5107**	Ecuador	Bolívar	Above Balzapamba	-1.83601, -79.13322	2026
*A.roulei*	**QCAZ 6256**	Ecuador	Azuay	Hierba Mala	-2.70430, -79.43367	2427
*A.roulei*	**ZSFQ 4943**	Ecuador	Chimborazo	Tixán	-2.16174, -78.81227	2892
*A.roulei*	**ZSFQ 4944**	Ecuador	Chimborazo	Tixán	-2.16174, -78.81227	2892
*A.roulei*	**ZSFQ 4942**	Ecuador	Chimborazo	Tixán	-2.16174, -78.81227	2892
*A.roulei*	**ZSFQ 4941**	Ecuador	Chimborazo	Tixán	-2.16174, -78.81227	2892
*A.roulei*	**ZSFQ 4940**	Ecuador	Chimborazo	Tixán	-2.16174, -78.81227	2892
*A.roulei*	**ZSFQ 4945**	Ecuador	Chimborazo	Tixán	-2.16174, -78.81227	2892
*A.zgap* sp. nov.	**ZSFQ 4946**	Ecuador	Napo	Santa Rosa	-0.31004, -77.78591	1500
*A.zgap* sp. nov.	**QCAZ 12666**	Ecuador	Napo	Borja, 1 km NE of	-0.40954, -77.84005	1703
*A.zgap* sp. nov.	**QCAZ 5183**	Ecuador	Napo	Bosque La Cascada	-0.14572, -77.49593	1460

### ﻿Sampling

Tissue samples from 12 individuals representing seven species (including the three new species described here) were obtained in Ecuador. All specimens included in the genetic analyses were morphologically identified according to Savage (1960), Arteaga et al. (2017), Melo-Sampaio et al. (2021), and Arteaga et al. (2022). We generated sequence data for samples marked with an asterisk under Appendix I, which includes museum vouchers at MZUTI, MZUA, and ZSFQ.

### ﻿Laboratory techniques

Genomic DNA was extracted from 96% ethanol-preserved tissue samples (liver, muscle tissue, or scales) using either a guanidinium isothiocyanate extraction protocol (Peñafiel et al. 2020), or a modified salt precipitation method based on the Puregene DNA purification kit (Gentra Systems). The nucleotide sequences of the primers and the PCR conditions applied to each primer pair are detailed in Appendix II. PCR products were cleaned with either ExoSAP-IT (Affymetrix, Cleveland, OH), or Exonuclease I and Alkaline Phosphatase (Illustra ExoProStar by GE Healthcare) before they were sent to Macrogen Inc (Seoul, South Korea) for sequencing. All PCR products were sequenced in both forward and reverse directions with the same primers that were used for amplification. The edited sequences were deposited in GenBank (Appendix I).

### ﻿DNA phylogenetic analyses

A total of 274 DNA sequences were used to build a phylogenetic tree of the genus *Atractus*, of which 32 were generated during this work and 242 were downloaded from GenBank, most of which were produced by Arteaga et al. (2017), Melo-Sampaio et al. (2021), and Passos et al. (2022). Of these, 85 sequences are 367–516 bp long fragments of the 16S gene, 66 are 578–1,079 bp long fragments of the CYTB gene, 69 are 567–849 bp long fragments of the ND4 gene, 18 are 513–573 bp long fragments of the C-MOS gene, 19 are 386–516 bp long fragments of the NT3 gene, and 17 are 736 bp long fragments of the RAG-1 gene. New sequences were edited and assembled using the program Geneious ProTM 2021.1.1 (Drummond et al. 2021) and aligned with those downloaded from GenBank (Appendix I) using MAFFT v.7 (Katoh and Standley 2013) under the default parameters in Geneious ProTM 2021.1.1. Genes were combined into a single matrix with 16 partitions, one per non-coding gene and three per protein coding gene corresponding to each codon position. The best partition strategies along with the best-fit models of evolution were obtained in PartitionFinder 2.1.1 (Lanfear et al. 2016) under the Bayesian information criterion.

Phylogenetic relationships were assessed under both a Bayesian inference (**BI**) approach in MrBayes 3.2.0 (Ronquist and Huelsenbeck 2013) and a maximum likelihood (**ML**) approach in RAxML-NG v. 1.1.0 (Kozlov et al. 2019). For the ML analysis, nodal support was assessed using the *standard* bootstrapping algorithm with 1000 non-parametric bootstraps. For the BI analysis, four independent analyses were performed to reduce the chance of converging on a local optimum. Each analysis consisted of 6,666,667 generations and four Markov chains with default heating settings. Trees were sampled every 1,000 generations and 25% of them were arbitrarily discarded as ‘‘burn-in.” The resulting 5,000 saved trees per analysis were used to calculate posterior probabilities (PP) for each bipartition in a 50% majority-rule consensus tree. We used Tracer 1.7.2 (Rambaut et al. 2022) to assess convergence and effective sample sizes (ESS) for all parameters. Additionally, we verified that the average standard deviation of split frequencies between chains and the potential scale reduction factor (**PSRF**) of all the estimated parameters approached values of ≤ 0.01 and 1, respectively. Genetic distances between *Atractusroulei* Despax, 1910 and its sister species were calculated using the uncorrected distance matrix in Geneious ProTM 2021.1.1. GenBank accession numbers are listed in Appendix I.

### ﻿Distribution maps and ecological niche models

We present ranges of occurrence for five species of *Atractus*, including the three new species described here. Presence localities are derived from museum vouchers (Table 1), photographic records (iNaturalist), and the literature (all summarized under Suppl. material 1: Table S1). For three of the five species, a binary environmental niche model (ENM) accompanies the dot maps. These models estimate potential areas of distribution on the basis of observed presences and a set of environmental predictors (Elith and Leathwick 2009). To delimit the occupancy areas and the potential species distribution, we used the BAM diagram proposal (Soberón and Peterson 2005; Peterson et al. 2011). To create the models, we used presence localities listed under Suppl. material 1: Table S1, 19 bioclimatic variables from Worldclim 1.4 (Hijmans et al. 2005), and Maxent 3.4.1k, an algorithm based on the principle of maximum entropy (Phillips et al. 2006; Elith et al. 2011; Renner and Warton 2013).

For the first explorative exercise, we used the 19 climate layers from the WorldClim project and assessed which variables were the most important for the model, according to the Jackknife test calculated in MaxEnt (Royle et al. 2012). Correlated environmental variables (r < 0.8) were identified using the PEARSON correlation test of PAST 3. In a second modelling exercise, we used the locality records for each species (Suppl. material 1: Table S1) and the variables identified in the first approach to generate the species distribution. 5,000 iterations were specified to the program with clamping and no extrapolation. All other parameters in MaxEnt were maintained at default settings. To create the binary environmental niche models, suitable areas were distinguished from unsuitable areas by setting a *minimum training presence* threshold value. The logistic format was used to obtain the values for habitat suitability (continuous probability from 0 to 1), which were subsequently converted to binary presence-absence values on the basis of the established threshold value, defined herein as *the minimum training presence*. The convergence threshold was set to 10^-5^, maximum iterations to 500, and the regularization parameter to “auto”.

## ﻿Results

### ﻿Molecular phylogeny and taxonomic consequences

**Figure 1.**
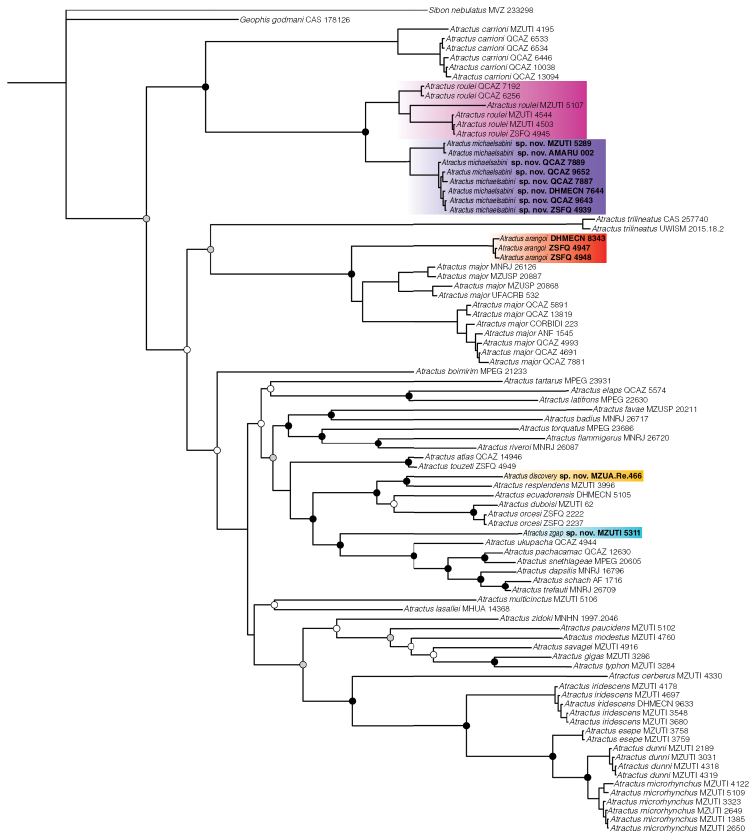
Phylogenetic relationships within *Atractus* inferred using a Bayesian inference and derived from analysis of 3,985 bp of DNA (gene fragments 16S, CYTB, ND4, C-MOS, NT3, and RAG1). Support values on intra-specific branches are not shown for clarity. Voucher numbers for sequences are indicated for each terminal. Black dots indicate clades with posterior probability values from 95–100%. Grey dots indicate values from 70–94%. White dots indicate values from 50–69% (values < 50% not shown). Colored clades correspond to the species’ distribution presented in the map of Fig. 2. New or resurrected species are indicated in bold type.

Selected partitions and models of evolution are presented in Table 2. We consider strong support for a clade when Bayesian analyses yield posterior probability values > 95%, following Felsenstein (2004), or when bootstrap values are greater than 70%. The overall topology and support of the BI (Fig. 1) and ML (Suppl. material 2: Figure S1) analyses are similar to that of Arteaga et al. (2017) and Passos et al. (2022). Species of the *Atractusroulei* species group are sister to all other sampled *Atractus* in the BI analysis, a view contrary to the ML analysis and to Murphy et al. (2019), in which *A.trilineatus* Wagler, 1928 and *A.boimirim*Passos et al., 2016, respectively are recovered as sister to all other *Atractus*. Below, we outline some differences between our analysis and those published in Murphy et al. (2019) and Passos et al. (2022).

**Table 2.** Partition scheme and models of evolution used in phylogenetic analyses. Numbers in parentheses indicate codon position.

Partition	Best model	Gene regions	Number of aligned sites
1	GTR+I+G	16S, cytb(3), ND4(1), NT3(1)	1202
2	HKY+I+G	cytb(1), ND4(2)	631
3	GTR+I+G	cytb(2), ND4(3)	630
4	JC	CMOS(1), NT3(3)	305
5	K80+I	CMOS(2), NT3(2), RAG1(2), RAG1(3)	794
6	HKY	CMOS(3), RAG1(1)	423

**Figure 2.**
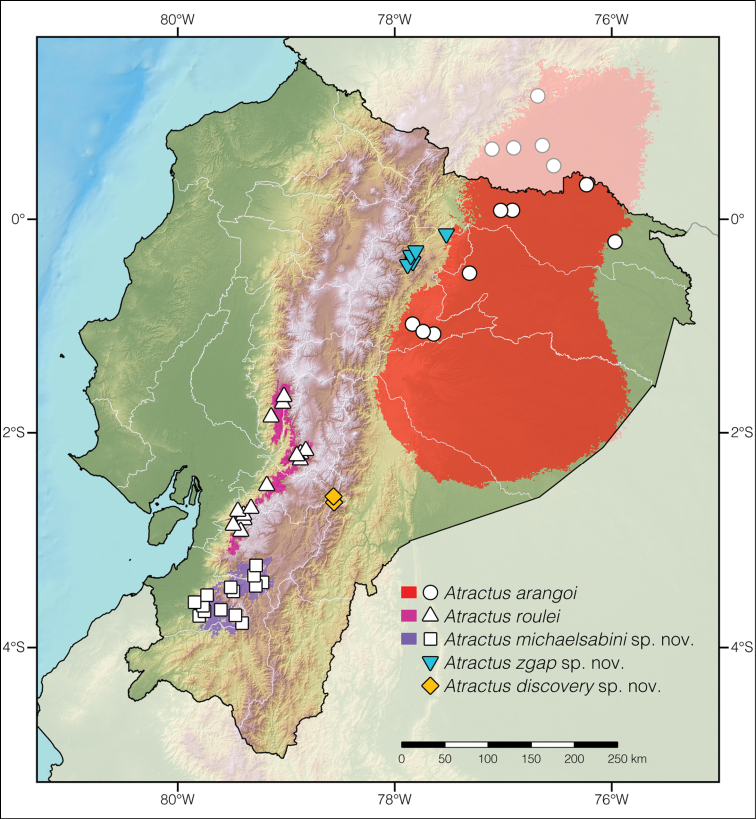
Distribution of *Atractusarangoi*, *A.roulei*, *A.michaelsabini* sp. nov., *A.zgap* sp. nov., and *A.discovery* sp. nov. in Ecuador and adjacent Colombia. White dots represent localities listed under Suppl. material 1. Each colored area is a geographic representation of the suitable environmental conditions for one of the clades recovered in the phylogeny of Fig. 1.

*Atractusroulei* is the strongly supported sister species of *A.carrioni* Parker, 1930, a relationship recovered in previous studies, but we found additional geographically structured genetic divergence within the former species (Figs 1, 2). We found moderate support for the placement of *A.trilineatus* as sister to *A.major* sensu Schargel et al. (2013), but strong support for the reciprocal monophyly between snakes assignable to *A.arangoi*, previously subsumed under *A.major*, and all other samples of *A.major*, including samples from throughout the species’ area of distribution. Samples labeled *A.arangoi* in our phylogeny are not closely related to *A.torquatus* (Duméril, Bibron, & Duméril, 1854), a name that has been applied to Ecuadorian specimens of the former (see Maynard et al. 2017). Our sample of *A.touzeti*Schargel et al., 2013 from the type locality is strongly supported as sister to the sample of *A.atlas*Passos et al., 2018. We found strong support for the relationship between *A.resplendens* Werner, 1901 from near the type locality and a new species from southeastern Ecuador. Our included samples of *A.orcesi* Savage, 1955 form a strongly supported sister clade to *A.duboisi* (Boulenger, 1880). A new species previously confused with *A.ecuadorensis* Savage, 1955, *A.orcesi*, and *A.resplendens* is not closely related to any of these species, but is recovered as the strongly supported sister species to a clade that contains *A.ukupacha*Melo-Sampaio et al., 2021, *A.pachacamac*Melo-Sampaio et al., 2021, *A.snethlageae* da Cunha & do Nascimento, 1983, *A.dapsilis*Melo-Sampaio et al., 2019, *A.schach* (Boie, 1827), and *A.trefauti*Melo-Sampaio et al., 2019. The latter two are sister species and their topological distance is smaller than intraspecific distances in other *Atractus* species sampled.

We find strong support for the relationship between members of the *Atractusiridescens* species group, which mirrors the results of Arteaga et al. (2017) and Murphy et al. (2019), and even those of Passos et al. (2022), although in the latter work some the terminals have been renamed. However, in the ML analysis (Suppl. material 2: Figure S1), *A.dunni* Savage, 1955 is weakly nested within *A.microrhynchus* Cope, 1868. Finally, we excluded *A.imperfectus* Myers, 2003 (voucher CH 9399) from the analyses as the short sequence available for comparison in GenBank (gene fragment 16S) represented a rogue taxon that assumed varying phylogenetic positions in the tree collection used to build the consensus tree.

### ﻿Systematic accounts

We name or provide redescriptions only for species that are monophyletic in our molecular phylogeny and share diagnostic features of their coloration pattern and lepidosis. Based on these species’ delimitation criteria, which follow the general species concept of de Queiroz (2007), we describe three new species of *Atractus*.

#### ﻿ Atractus discovery sp. nov.

Taxon classificationAnimaliaSquamataColubridae

B4E06A0D-FC6B-5164-9B85-3AEBD85F1CE6

https://zoobank.org/0343A95C-BC4B-4654-8333-55D8A34CD2EF

**Figure 3.**
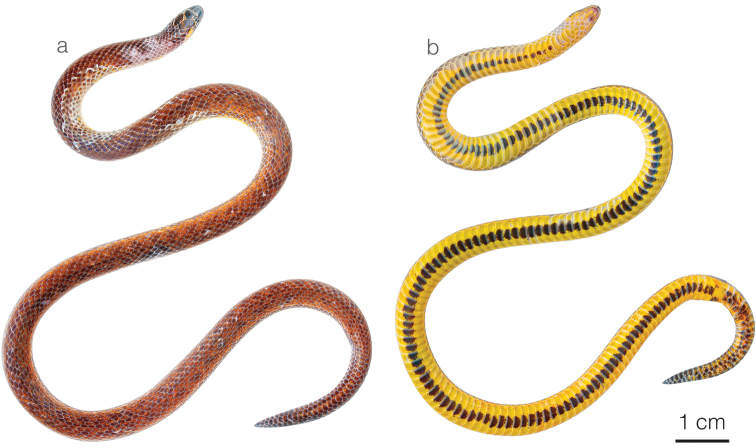
Adult male holotype of *Atractusdiscovery* sp. nov. ZSFQ 4937 in **a** dorsal and **b** ventral view.

**Figure 4.**
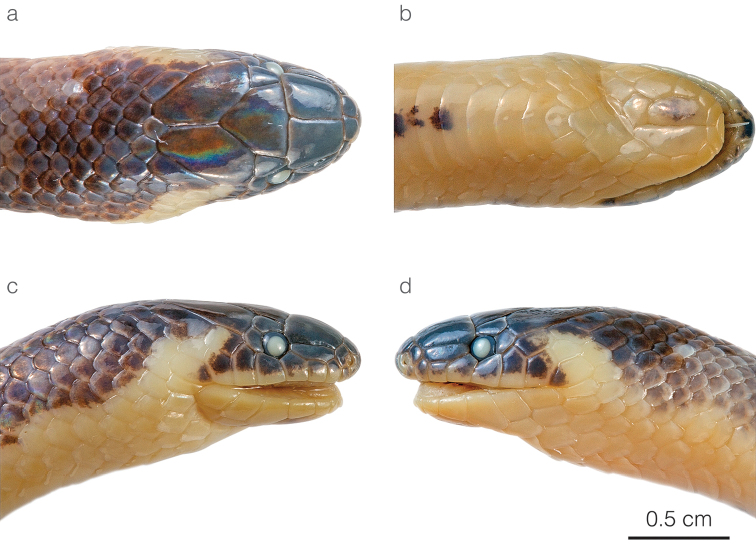
Head of the adult male holotype of *Atractusdiscovery* sp. nov. ZSFQ 4937 in **a** dorsal **b** ventral **c** lateral right, and **d** lateral left view.

**Figure 5.**
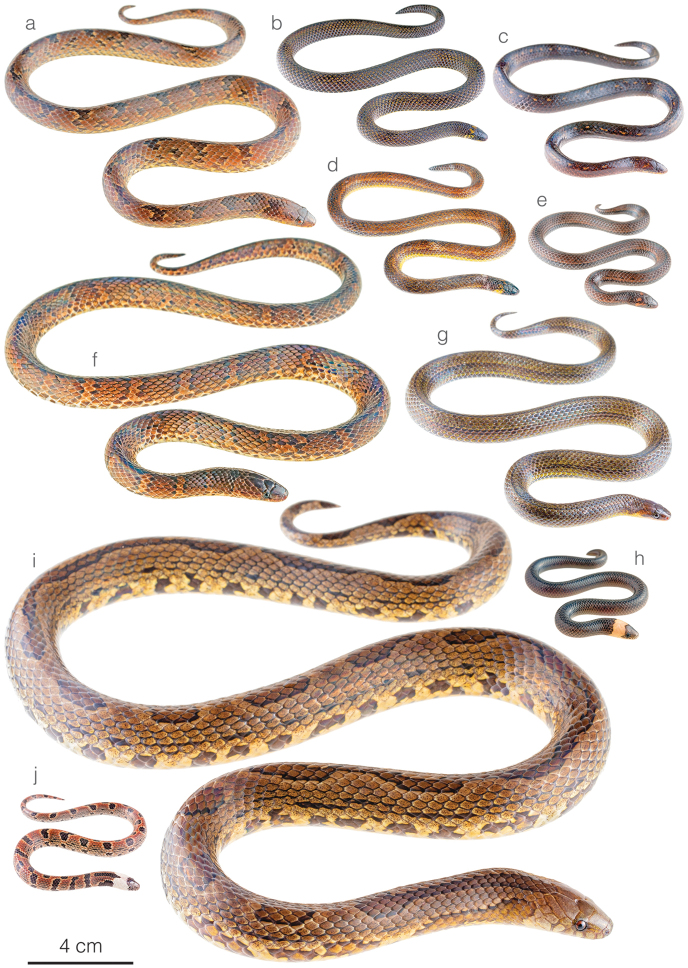
Photographs of living specimens of brown-colored *Atractus* occurring along the Amazonian slopes of the Andes in Ecuador **a***A.arangoi*ZSFQ 4948 from Jatun Sacha Biological Reserve, Napo province, Ecuador **b***A.resplendens*ZSFQ 4953 from Montañas de San Antonio, Tungurahua province, Ecuador **c***A.duboisi* from Orito Yacu, Napo province, Ecuador **d***A.discovery* sp. nov. ZSFQ 4936 from Amaluza, Azuay province, Ecuador **e***A.orcesi*ZSFQ 2234 from El Higuerón, Sucumbíos province, Ecuador **f***A.pachacamac* from Nangaritza, Zamora Chinchipe province, Ecuador **g***A.zgap* sp. nov. ZSFQ 4946 from Santa Rosa, Napo province, Ecuador **h***A.occipitoalbus* JMG-2077 from Macas, Morona Santiago province, Ecuador **i***A.major* from Jatun Sacha Biological Reserve, Napo province, Ecuador; and **j***A.major* from Reserva Natural Palmarí, Amazonas state, Brazil (photo by Sebastián Di Doménico).

Figs 3 , 4 , 5d Proposed standard english name: Discovery Ground Snake. Proposed standard spanish name: Culebra tierrera de Discovery.

##### Holotype.

ZSFQ 4937 (Figs 3, 4), adult male collected by Alejandro Arteaga and Amanda Quezada at Amaluza, Azuay province, Ecuador (S2.61582, W78.56537; 2002 m).

##### Paratypes.

ZSFQ 4936 (Fig. 5d), adult female collected by Alejandro Arteaga and Amanda Quezada at the type locality. MZUA.Re.466, adult female collected on 16 November 2018 at Campamento Arenales, Morona Santiago province, Ecuador (S2.59253, W78.56507; 2057 m).

##### Diagnosis.

*Atractusdiscovery* sp. nov. is placed in the genus *Atractus*, as diagnosed by Savage (1960), based on phylogenetic evidence (Fig. 1). The species is diagnosed based on the following combination of characters: (1) 17/17/17 smooth dorsals; (2) one postocular; (3) loreal 2.5–3 × longer than high; (4) temporals 1+2; (5) eight supralabials, fourth and fifth contacting orbit; (6) seven infralabials, first four contacting chinshields; (7) six or seven maxillary teeth; (8) one row of gular scales; (9) three preventrals; (10) 168 ventrals in the male holotype (Fig. 3b) and 170–172 ventrals in females; (11) 27 subcaudals in the male holotype and 17–18 subcaudals in females; (12) dorsal ground color light brown with faint stippling of a darker shade (Figs 3a, 5d); (13) venter yellow with a brown ventral stripe (Fig. 3b); (14) 284 mm SVL in the male holotype and 308–328 mm SVL in females; (15) 28 mm TL in the male holotype and 19–24 mm TL in females.

##### Comparisons.

*Atractusdiscovery* sp. nov. differs from most of its congeners by having a bright yellow belly with a conspicuous dark brown longitudinal stripe. This species is compared to other small brownish congeneric ground snakes distributed along the Amazonian slopes of the Andes (most of these are pictured in Fig. 5): *Atractusavernus*Passos et al., 2009b, *A.duboisi*, *A.ecuadorensis*, *A.zgap* sp. nov., *A.occipitoalbus* (Jan, 1862), *A.orcesi*, and *A.resplendens*. From *A.avernus*, *A.duboisi*, *A.occipitoalbus*, and *A.orcesi*, the new species differs in having 17/17/17 (instead of 15/15/15) dorsal scale rows. From *A.ecuadorensis*, *A.zgap* sp. nov., and *A.resplendens*, it differs in having a bright yellow belly with a conspicuous dark brown longitudinal stripe. From *A.ecuadorensis* and *A.zgap* sp. nov., it further differs by having one (instead of two) postocular scale (Fig. 4c).

##### Description of holotype.

Adult male, SVL 284 mm, tail length 28 mm (9.9% SVL); body diameter 7.8 mm; head length 8.8 mm (3.1% SVL); head width 5.6 mm (2.0% SVL); interocular distance 3.4 mm; head slightly distinct from body; snout-orbit distance 3.4 mm; rostral 1.6 mm wide, ca. as broad as high; internasals 0.9 mm wide; prefrontals 2.1 mm wide; frontal 2.9 mm wide, with a curvilinear triangular shape in dorsal view; parietals 2.2 mm wide, ~ 2 × as long as wide; nasal divided; loreal 2.0 mm long, ~ 3 × longer than high; eye diameter 1.1 mm; pupil round; supraoculars 1.3 mm wide; one postocular; temporals 1+2, upper posterior temporal elongate; eight supralabials, fourth and fifth contacting orbit; symphysial 1.0 mm wide, ~ 2 × as broad as long and separated from chinshields by first pair of infralabials; seven infralabials, first four contacting chinshields; chinshields ~ 2 × as long as broad, posterior chinshields absent; four rows of gular scales; dorsal scales arranged in 17/17/17 rows, smooth without apical pits; two preventrals; ventrals 168; anal plate single; 27 paired subcaudals.

##### Natural history.

The three known specimens of *Atractusdiscovery* sp. nov. were found in open areas adjacent to cloud forest border. MZUA.Re.466 was crawling at ground level at around 7:30 pm. It was crossing a series of cement stairs. ZSFQ 4936 and ZSFQ 4937 were found during a cloudy day, buried 15–40 cm under soft soil at the border between the clearing of a graveyard, pastures, and remnants of native vegetation.

##### Distribution.

*Atractusdiscovery* sp. nov. is known only from two localities (Arena­les and Amaluza, listed under Suppl. material 1: Table S1) on each side of the Río Paute, in the Ecuadorian provinces Azuay and Morona Santiago, at elevations 2002–2057 m a.s.l. The airline distance between the two localities is 2.6 km (Fig. 2).

##### Etymology.

The specific epithet *discovery* is used as a noun in apposition and honors ‘The Explorers Club Discovery Expedition Grants’ (https://www.explorers.org/grants) initiative, a program seeking to foster scientific understanding for the betterment of humanity and all life on Earth and beyond. The grant program supports researchers and explorers from around the world in their quest to mitigate climate change, prevent the extinction of species and cultures, and ensure the health of the Earth and its inhabitants. ‘The Explorers Club Discovery Expedition Grants’ program funded the expedition that resulted in the discovery of this new species of snake.

##### Conservation status.

We consider *Atractusdiscovery* sp. nov. to be Data Deficient, following IUCN Red List criteria, because the species belongs to a poorly studied genus of snakes and is known only from three specimens collected recently in a single river valley (Río Paute) in the Amazonian slopes of the Ecuadorian Andes. In addition to the presence of a system of major hydroelectric dams in this valley, most of the native cloud forest habitat in the segment between Amaluza and Arenales has been converted to pastures. However, we consider there is insufficient data to estimate whether this new snake species is restricted to the immediate environs of the type locality or if it is widely distributed along the unexplored cloud forests of the adjacent Sangay National Park.

#### ﻿ Atractus zgap sp. nov.

Taxon classificationAnimaliaSquamataColubridae

A21843AD-A6ED-5B1E-8FE0-46349F312062

https://zoobank.org/A9A58D40-CF58-4267-A691-B5E776B43C1B

**Figure 6.**
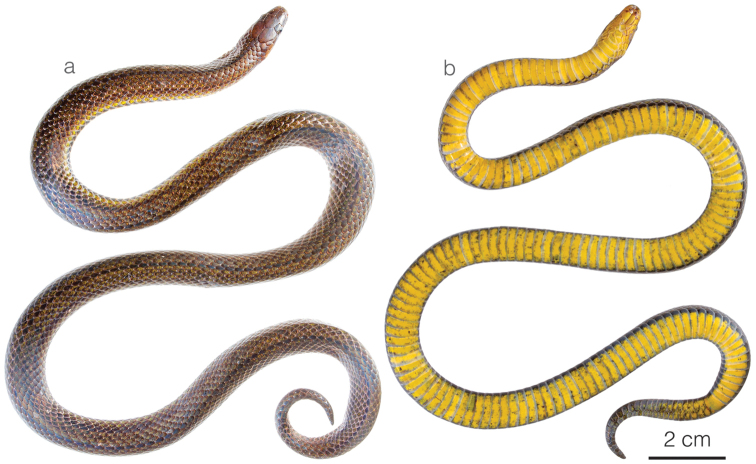
Adult female holotype of *Atractuszgap* sp. nov. ZSFQ 4946 in **a** dorsal and **b** ventral view.

**Figure 7.**
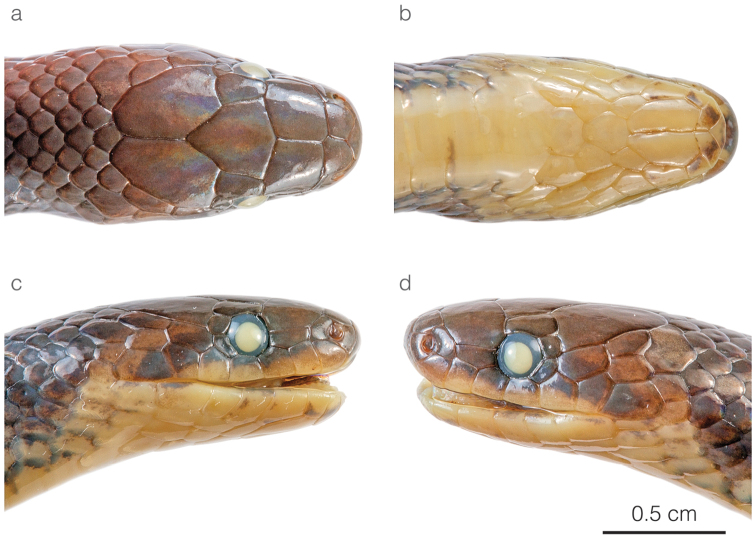
Head of the adult female holotype of *Atractuszgap* sp. nov. ZSFQ 4946 in **a** dorsal **b** ventral **c** lateral right, and **d** lateral left view.

Figs 5g , 6 , 7 Proposed standard English name: ZGAP Ground Snake. Proposed standard Spanish name: Culebra tierrera de ZGAP.

##### Holotype.

ZSFQ 4946 (Figs 5g, 6, 7), adult female collected by Diego Piñán at Santa Rosa, Napo province, Ecuador (S0.31004, W77.78591; 1500 m).

##### Paratypes.

MZUTI 5311, adult female collected by Diego Piñán in February 2017 at El Chaco, Napo Province, Ecuador (S0.31004, W77.78591; 1500 m). QCAZ 12666, a juvenile collected by Pablo Medrano on 16 May 2014 at San Francisco de Borja, Napo province, Ecuador (S0.40953, W77.84005; 1703 m). QCAZ 5183, a juvenile collected by Patricia Bejarano on 13 November 2011 at Bosque Protector “La Cascada,” Napo province, Ecuador (S0.14572, W77.49593; 1460 m).

##### Diagnosis.

*Atractuszgap* sp. nov. is placed in the genus *Atractus*, as diagnosed by Savage (1960), based on phylogenetic evidence (Fig. 1). The species is diagnosed based on the following combination of characters: (1) 17/17/17 smooth dorsals; (2) two postoculars; (3) loreal 2 × longer than high; (4) temporals 1+2; (5) seven supralabials, third and fourth contacting orbit; (6) seven infralabials, first three contacting chinshields; (7) seven maxillary teeth; (8) three rows of gular scales; (9) two or three preventrals; (10) 173–177 ventrals in females; (11) 31 subcaudals in an uncollected male and 25–27 subcaudals in females; (12) dorsal ground color brown with faint dark longitudinal lines (Figs 5g, 6a); (13) venter yellow with fine brown stippling (Fig. 6b); (14) 376 mm SVL in the female holotype; (15) 37 mm TL in the female holotype.

##### Comparisons.

*Atractuszgap* sp. nov. is compared to other small brownish congeneric ground snakes distributed along the Amazonian slopes of the Andes (most of these are illustrated in Fig. 5): *Atractusavernus*, *A.duboisi*, *A.discovery* sp. nov., *A.ecuadorensis*, *A.occipitoalbus*, *A.orcesi*, and *A.resplendens*. From *A.avernus*, *A.duboisi*, *A.occipitoalbus*, and *A.orcesi*, the new species differs in having 17/17/17 dorsal scale rows. From *A.discovery* sp. nov., the new species differs in having two postocular scales (Fig. 7c) and no dark ventral stripe. From *A.ecuadorensis*, the new species differs in having fewer (31 instead of 41) subcaudals in males, seven (instead of five or six) infralabials, a shorter (2 × instead of 3 × longer than high) loreal, frontal longer than prefrontals, and five faint (instead of six or seven clearly defined) longitudinal black lines (Figs 5g, 6). From *A.resplendens*, the new species differs in having a shorter (2 × instead of 3 × longer than high) loreal, two (instead of one) postoculars, and a brownish dorsum with faint longitudinal black lines, whereas in *A.resplendens* the dorsum is dark gray with fine yellow stippling (Fig. 5b).

##### Description of holotype.

Adult female, SVL 376 mm, tail length 37 mm (9.8% SVL); body diameter 9.1 mm; head length 11.7 mm (3.1% SVL); head width 6.4 mm (1.7% SVL); interocular distance 4.3 mm; head slightly distinct from body; snout-orbit distance 3.8 mm; rostral 2.5 mm wide, ca. as broad as high; internasals 1.3 mm wide; prefrontals 2.5 mm wide; frontal 3.1 mm wide, with a curvilinear triangular shape in dorsal view; parietals 2.4 mm wide (56% length); nasal divided; loreal 1.6 mm long, ~ 2 × longer than high; eye diameter 1.7 mm; pupil round; supraoculars 1.2 mm wide; two postoculars; temporals 1+2; seven supralabials, third and fourth contacting orbit; symphysial 1.7 mm wide, ~ 2 × as broad as long, separated from chinshields by first pair of infralabials; seven infralabials, first three contacting chin shields; chinshields ~ 2 × as long as broad, posterior chinshields absent; dorsal scales arranged in 17/17/17 rows, smooth without apical pits; two preventrals; ventrals 173; anal plate single; 25 paired subcaudals.

##### Natural history.

Most individuals of *Atractuszgap* sp. nov. have been found during the day hidden under rocks, among herbs, or buried under soft soil in plantations and rural gardens close to remnants of native forest. At night, they have been seen crossing rural roads. Occasionally, during sunny days right after a rain, individuals have been seen crawling on the pavement or on gravel roads (Diego Piñán, pers. comm.).

##### Distribution.

*Atractuszgap* sp. nov. is known only from five localities (See Suppl. material 1: Table S1) along the valley of the Río Quijos, Napo province, in the Amazonian slopes of the Andes in northeastern Ecuador, at elevations 1460–1703 m a.s.l. (Fig. 2).

##### Etymology.

The specific epithet *zgap* is used as a noun in apposition and honors the ‘Zoological Society for the Conservation of Species and Populations’ (ZGAP) (https://www.zgap.de), a program seeking to conserve unknown but highly endangered species and their natural habitats throughout the world. The ZGAP grant program supports the fieldwork of young scientists who are eager to implement and start conservation projects in their home countries. Specifically, ZGAP has supported the work on endangered Andean reptiles in Ecuador conducted by AA and JV.

##### Conservation status.

We consider *Atractuszgap* sp. nov. to be Endangered following the IUCN criteria B2a, b (i, iii) (IUCN 2001), because the species’ extent of occurrence is estimated to be less than 500 km^2^ (Fig. 2) and its habitat is severely fragmented and declining in extent and quality due to deforestation. The valley of the Río Quijos formed the eastern frontier of the Incan Empire (1400–1532) and the cloud forest in the area suffered from intensive land-use even before European arrival (Loughlin et al. 2018). Today, this valley is one of the most important cattle farming areas along the eastern slopes of the Andes and the majority of the forest along the Quijos river plains has been destroyed. Although *A.zgap* occurs in one protected area (Bosque Protector “La Cascada”) and its presence is expected in adjacent Parque Nacional Cayambe-Coca and Parque Nacional Sumaco Napo-Galeras, it has so far not been recorded in major protected areas.

#### ﻿ Atractus michaelsabini sp. nov.

Taxon classificationAnimaliaSquamataColubridae

40F34958-F8F5-5BD7-9218-C051526D04A4

https://zoobank.org/E85C68A2-DAEF-4BC5-A6B3-6D1FEDEB9983

**Figure 8.**
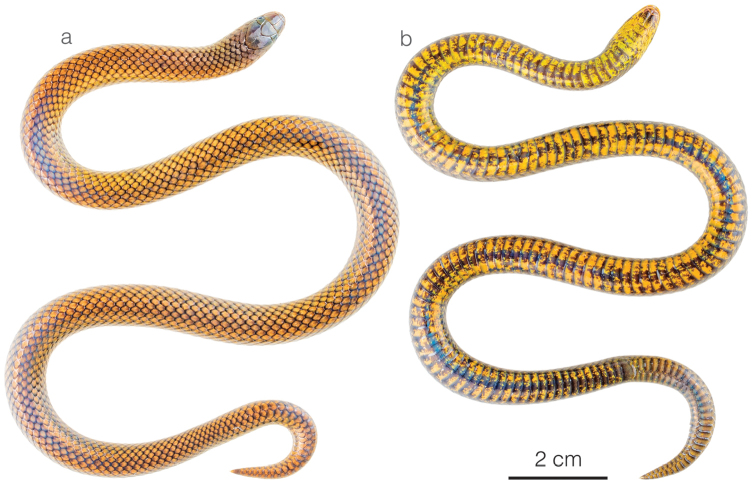
Adult male holotype of *Atractusmichaelsabini* sp. nov. ZSFQ 4938 in **a** dorsal and **b** ventral view.

**Figure 9.**
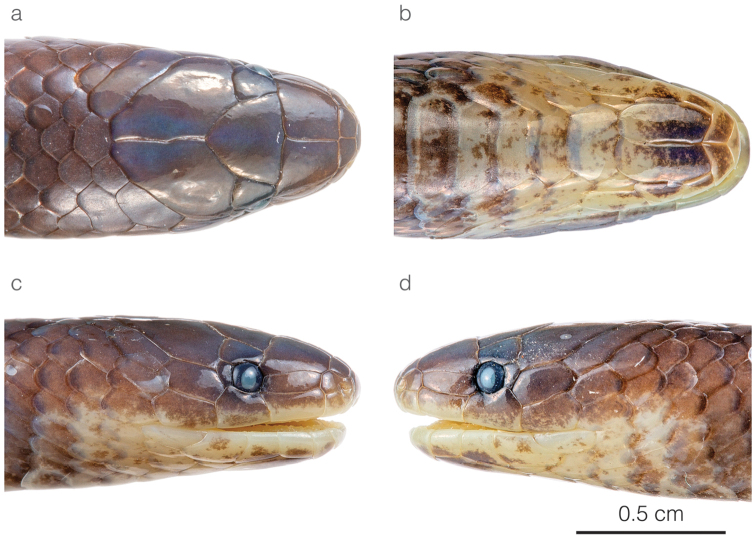
Head of the adult male holotype of *Atractusmichaelsabini* sp. nov. ZSFQ 4938 in **a** dorsal **b** ventral **c** lateral right, and **d** lateral left view.

**Figure 10.**
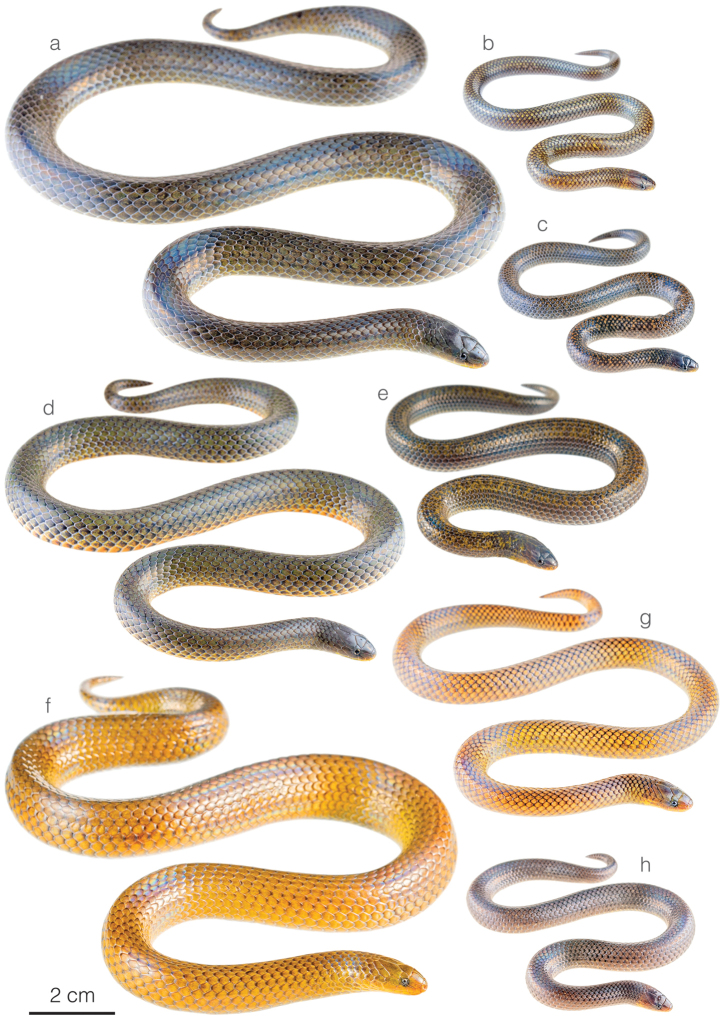
Photographs of living specimens of *Atractusroulei* and *A.michaelsabini* sp. nov. **a***A.roulei*ZSFQ 4942 from Tixán, Chimborazo province, Ecuador **b***A.roulei*ZSFQ 4944 from Tixán, Chimborazo province, Ecuador **c***A.roulei*ZSFQ 4941 from Tixán, Chimborazo province, Ecuador **d***A.roulei*ZSFQ 4945 from Tixán, Chimborazo province, Ecuador **e***A.roulei* from Tixán, Chimborazo province, Ecuador **f***A.michaelsabini* sp. nov. AMARU 002 from Corraleja, Azuay province, Ecuador **g***A.michaelsabini* sp. nov. holotype ZSFQ 4938 from Corraleja, Azuay province, Ecuador and **h***A.michaelsabini* sp. nov. ZSFQ 4939 from El Panecillo, El Oro province, Ecuador.

Figs 8 , 9 , 10f–h Proposed standard English name: Michael Sabin’s Ground Snake. Proposed standard Spanish name: Culebra tierrera de Michael Sabin.

Atractus roulei Savage, 1960: 68 (part).
Atractus lehmanni Arteaga et al., 2017: 97.

##### Holotype.

ZSFQ 4938 (Figs 8, 9, 10g), adult male collected by Jorge Luis Romero at Corraleja, Azuay province, Ecuador (S3.3874, W79.22785; 2660 m).

##### Paratypes.

MZUTI 5289, adult female collected by Jorge Luis Romero at the type locality. AMARU 002 (Fig. 10f), adult female collected by Jorge Luis Romero at the type locality. ZSFQ 4939 (Fig. 10h), juvenile female collected by Jose Vieira and Amanda Quezada at El Panecillo, El Oro province, Ecuador (S3.46753, W79.48248; 2750 m). QCAZ 7887 and 7902, adult male and female collected by Silvia Aldás in December 2006 at Guanazán, El Oro province, Ecuador (S3.44667, W79.49051; 2663 m). QCAZ 9643 and 9652, adult females collected by Silvia Aldás in August 2009 at El Panecillo, El Oro province, Ecuador (S3.46753, W79.48248; 2775 m). DHMECN 7644–45, adult males collected by Mario Yánez-Muñoz, Luis Oyagata, Patricia Bejarano, and Marco Altamirano in March 2010 at Reserva Biológica Yunguilla, Azuay province, Ecuador (S3.22684, W79.27520; 1748 m). AMNH 18325, adult female collected in July 1920 at El Chiral, El Oro province, Ecuador (S3.63825, W79.59723; 1841 m). AMNH 22110–11, collected in August 1921 at La Chonta, El Oro province, Ecuador (S3.56585, W79.85144; 1025 m).

##### Diagnosis.

*Atractusmichaelsabini* sp. nov. is placed in the genus *Atractus*, as diagnosed by Savage (1960), based on phylogenetic evidence (Fig. 1). The species is diagnosed based on the following combination of characters: (1) 15/15/15 smooth dorsals; (2) one postocular; (3) loreal 3 × longer than high; (4) temporals 1+2; (5) five or six supralabials, with (usually) third and fourth contacting orbit; (6) five or six infralabials, with (usually) first three contacting chinshields; (7) 9–13 maxillary teeth; (8) 1–3 rows of gular scales; (9) 1–3 preventrals; (10) 143–144 ventrals in males and 144–153 in females; (11) 24–31 subcaudals in males and 17–19 in females; (12) dorsal ground color golden yellow (Figs 8, 10f–g) to dark brown (Fig. 10h) with each scale outlined in black, forming a reticulation; (13) venter yellowish with various degrees of brown stippling (Fig. 8b); (14) 256–321 mm SVL in males and 201–392 mm SVL in females; (15) 35–42 mm TL in males and 21–37 mm TL in females.

##### Comparisons.

*Atractusmichaelsabini* sp. nov. is compared to other members of the *A.roulei* species group: *Atractuscarrioni* and *A.roulei*. From *A.carrioni*, the new species differs in having a loreal scale (Fig. 9c) (absent in *A.carrioni*). From *A.roulei* (Figs 10a–e), the new species differs in having a dorsal pattern in which each scale is outlined in a thin black line, thus creating a reticulation, and by having the prefrontal scale in broad contact with the postnasal (Fig. 9c) (not in contact or barely in contact in *A.roulei*). Furthermore, the existence of the bright golden yellow morph in adult individuals has so far been recorded only in *A.michaelsabini* sp. nov.; not in *A.roulei*, where adults are dark brown dorsally (Fig. 10a–e). In *A.roulei*, there is a black spot at the base of each dorsal scale, whereas in *A.michaelsabini* sp. nov. the spot is at the tip of each dorsal scale and is connected to the black reticulum. Genetic divergence in a 578 bp long fragment of the mitochondrial CYTB gene between *A.michaelsabini* sp. nov. and *A.roulei* is 6.5–7.2%, whereas intraspecific distances are 0–4.5% in *A.michaelsabini* sp. nov. and 0–4.8% in *A.roulei*.

##### Description of holotype.

Adult male, SVL 256 mm, tail length 39 mm (15.2% SVL); body diameter 7.4 mm; head length 10.7 mm (3.1% SVL); head width 6.4 mm (2.5% SVL); interocular distance 3.7 mm; head slightly distinct from body; snout-orbit distance 3.5 mm; rostral 1.9 mm wide, ca. as broad as high; internasals 1.0 mm wide; prefrontals 2.0 mm wide; frontal 3.0 mm wide, with a curvilinear triangular shape in dorsal view; parietals 2.9 mm wide (65% length); nasal divided; loreal 2.2 mm long, ~ 3 × longer than high; eye diameter 1.4 mm; pupil round; supraoculars 1.3 mm wide; one postocular; temporals 1+2; five supralabials, third contacting orbit; symphysial 1.7 mm wide, ~ 3 × as broad as long, separated from chinshields by first pair of infralabials; five infralabials, first three contacting chinshields; chinshields ~ 2 × as long as broad, posterior chinshields absent; dorsal scales arranged in 15/15/15 rows, smooth without apical pits; no preventrals; ventrals 143; anal plate single; 31 paired subcaudals.

##### Natural history.

Most individuals of *Atractusmichaelsabini* sp. nov. have been found during the day hidden under rocks, mats of rotten vegetation, or buried in soft soil in pastures and maize plantations close to remnants of native forest. At night, they have been seen crossing forest trails. At the type locality, clutches of three or four eggs have been found under soil (Jorge Luis Romero, pers. comm.). Anecdotal information suggests that these snakes are more active during the rainy months (February-May at the type locality; Jorge Luis Romero, pers. comm.).

##### Distribution.

*Atractusmichaelsabini* sp. nov. is endemic to an estimated 2,530 km^2^ area along the Pacific slopes of the Andes in southwestern Ecuador. The species occurs in the xeric inter-Andean valley of the Río Jubones as well as on the slopes of the Cordillera de Chilla. *Atractusmichaelsabini* sp. nov. is known from provinces Azuay, El Oro, and Loja, and has been recorded at elevations between 927 and 2922 a.s.l. (Fig. 2).

##### Etymology.

The specific epithet *michaelsabini* is a patronym honoring a young nature lover, Michael Sabin, grandson of American philanthropist and conservationist Andrew “Andy” Sabin. The Sabin family is involved in conservation and field research of amphibians and reptiles and has protected over 264,365 acres of critical habitat throughout the world.

##### Conservation status.

We consider *Atractusmichaelsabini* sp. nov. to be Endangered following the IUCN criteria B1a, b (i, iii) (IUCN 2001), because the species’ extent of occurrence is estimated to be much less than 5,000 km^2^ (Fig. 2) and its habitat is severely fragmented and declining in extent and quality due to deforestation. Although *A.michaelsabini* sp. nov. is present in two protected areas (private reserves Buenaventura and Yunguilla of Fundación Jocotoco), nine of the 14 localities where the species has been recorded (Suppl. material 1: Table S1) are in heavily human-modified areas. Based on maps of Ecuador’s vegetation cover (MAE 2012), we estimate that nearly 70% of the forest cover throughout the species’ potential distribution area has been destroyed, mostly due to the expansion of the agricultural frontier.

### ﻿Distribution maps

Our resulting distribution maps increase the number of known localities of occurrence for the studied taxa (listed under Suppl. material 1: Table S1) and show a distinct geographical separation between *Atractusroulei* and *A.michaelsabini* sp. nov. (Fig. 2). The predicted area of suitable habitat for *A.michaelsabini* sp. nov. includes the upper watershed of the Río Jubones (a xeric inter-Andean valley) as well as both slopes of the Cordillera de Chilla (an area having vegetation classified as evergreen montane forest; see Sierra 1999). Likewise, the predicted area of suitable habitat for *A.roulei* includes evergreen montane forests along the Pacific slopes of the Andes as well as the xeric inter-Andean valley of the upper Río Chanchán. The predicted area of suitable habitat for *A.arangoi* includes almost the entire extent of Pastaza province, although we did not find records of this species from this province. Although we did not build binary environmental niche models for *A.discovery* sp. nov. and *A.zgap* sp. nov. (only two and six localities are available for these species), they are both known only from their corresponding river valleys and occur on both sides of the Río Paute and Río Quijos, respectively.

### ﻿Revalidation of *Atractusarangoi*

**Figure 11.**
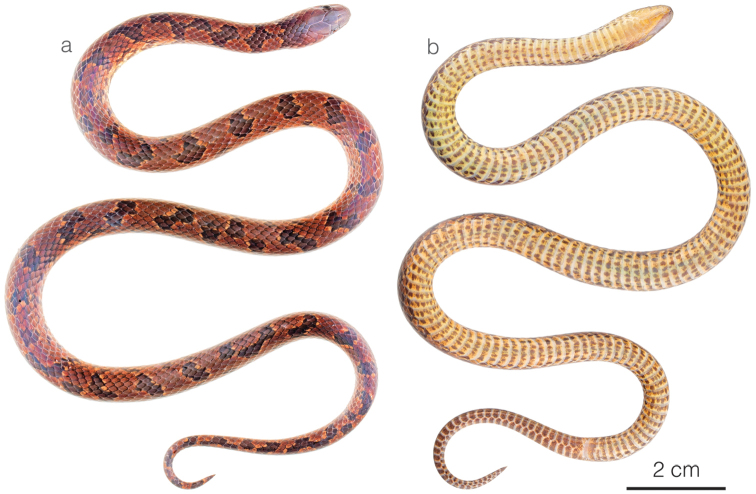
Adult male of *Atractusarangoi*ZSFQ 4947 in **a** dorsal and **b** ventral view.

Prado (1939) described *Atractusarangoi* from Colombia whereas Daniel (1949) reported this species in Puerto Asís, Putumayo department. Schargel et al. (2013) considered *A.arangoi* to be a junior synonym of *A.major* claiming that all the putative diagnostic characters for *A.arangoi* fall within the variation in *A.major* as defined in their work. In our phylogenetic tree of *Atractus* (Fig. 1), we included sequences of three snakes that fit the original description of *A.arangoi*. DHMECN 8343 (reported as *A.major* in Arteaga et al. 2017), ZSFQ 4947 (Fig. 11), and ZSFQ 4948 (Fig. 5a). These three specimens form a strongly supported clade sister to all other samples of *A.major*, which includes specimens from throughout the latter species’ area of distribution. Furthermore, we find that these specimens, in addition to others reported in the literature as *A.torquatus* and *A.major* (see Duellman 1978; Maynard et al. 2017) can easily be separated from *A.major* based on differences in coloration, body size (compare Figs 5a and 5i, j), and ventral and subcaudal counts (summarized in Table 3), as originally suggested by Prado (1939). Thus, we formally remove *A.arangoi* from the synonymy of *A.major*, include this species in the herpetofauna of Ecuador, and provide a distribution map for this species (Fig. 2).

**Table 3.** Differences in coloration, scale counts, and size between *Atractusarangoi* and *A.major*. The range of each continuous variable is from our own sample, Prado (1939), and Maynard et al. (2017). The numbers in parentheses represent the sample size.

Variable character	*Atractusarangoi*		*Atractusmajor*	
Dark brown or black nape stripe	Absent		Present	
Dorsal markings	Irregular dark blotches		Complete irregular dark bands anteriorly; blotches posteriorly	
Sex	Males (*n* = 2)	Females (*n* = 2)	Males (*n* = 7)	Females (*n* = 5)
Maximum SVL	309 mm	412 mm	533 mm	986 mm
Ventral scales	154–163	160–161	162–165	172–177
Subcaudal scales	38–39	29–32	36–45	34–37

### ﻿Presence of *Atractusgigas* in Peru

Passos et al. (2010) reported *Atractusgigas*, a snake species previously considered to be endemic to the cloud forests of northwestern Ecuador (Myers and Schargel 2006), on the Amazonian slopes of the Andes in Peru. The identification of the Peruvian specimens as *A.gigas* was based on their large size and the partial overlap in some characters of lepidosis with the Ecuadorian samples. However, these Peruvian snakes have a smaller number of subcaudals (25 or 26 instead of 31–37 in Ecuadorian specimens), a shorter loreal scale, first four infralabials contacting chinshields (instead of first three in Ecuadorian specimens), and a completely different color pattern in both juveniles and adults (for a figure depicting the variation among Ecuadorian individuals see Arteaga 2022). Juveniles of “*A.gigas*” from Peru have a black dorsum with short (one scale wide) reddish brown bands whereas juveniles of Ecuadorian *A.gigas* have a contrasting pattern of dark-brown to black rounded bands or blotches on a rosy white background color. Adults of “*A.gigas*” from Peru have a dorsal pattern in which each scale is dark brown distally but cream towards the base, forming a reticulation. Adults of *A.gigas* from Ecuador are uniformly rich dark brown or glossy black, and the skin between the scales is whitish (Arteaga 2022). QCAZ 14946, a specimen identified as *A.atlas* in Melo-Sampaio et al. (2021) from Reserva Biológica Cerro Plateado, just 7 km from the Peruvian border on the southeastern slopes of the Ecuadorian Andes, resembles Peruvian “*A.gigas*” as depicted in Passos et al. (2010) in having a short loreal, dorsal scales with a cream base, first four infralabials contacting chinshields, and fewer than 30 subcaudals. This specimen was included in our phylogeny (Fig. 1) and was recovered as the strongly supported sister taxon to a new sample of *A.touzeti* from this species’ type locality. Based on this evidence, we suggest that Peruvian specimens CORBIDI 877 and ZFMK 89147, as well as other *Atractus* specimens from Cajamarca labeled as *A.gigas*, be reidentified as *A.atlas*, or at the very least, be considered as an undescribed species related to the latter. Thus, we suggest *A.gigas* be removed from the herpetofauna of Peru, a view that confirms this species as endemic to the cloud forests of northwestern Ecuador as originally suggested by Myers and Schargel (2006) and Arteaga et al. (2013).

### ﻿Status of *Atractusoccidentalis* and reidentification of specimens of *Atractus* of the iridescens group

In his unpublished BSc thesis, Mejía Guerrero (2018) used species distribution models, a comprehensive (based on 88 specimens) comparison of scale counts, and species delimitation analysis based on a combination of novel DNA sequences and those provided in Arteaga et al. (2017) to test species limits within the *Atractusiridescens* species group. He proposed that *A.occidentalis* Savage, 1955 is a junior synonym of *A.microrhynchus* and that some individuals identified as *A.dunni* from Mindo are actually *A.microrhynchus*. The topology for the included members of the *A.iridescens* group in our BI phylogeny (Fig. 1) and that of Murphy et al. (2019), though not identical, agree with the proposal of Mejía Guerrero (2018). Based this evidence, we also consider *A.occidentalis* to be a junior synonym of *A.microrhynchus*. Recently, Passos et al. (2022) provided a list of reidentifications of 15 (not 17, because two are duplicates and MZUTI 4178 retained the same identification despite being listed in the table) *Atractus* specimens having sequences deposited in GenBank, notably among them the members of the *A.iridescens* species group deposited in MZUTI and DHMECN. In this work, one reidentification (that of the holotype of *A.pyroni*; MZUTI 5107) was backed up by ample evidence and two others (ANF 2390, now MZUTI 5409; and GFM 307, now MPEG 21582) were substantiated in Melo-Sampaio et al. (2021), but the remaining were proposed without providing any evidence, either in the form of new phylogenetic relationships, new scale counts, or previously unsampled morphological features. Since these specimens are deposited at MZUTI and DHMECN, as well as their corresponding photo vouchers available in Arteaga et al. (2017), and their DNA sequences on GenBank, their identity can be tested by anyone. Although the reidentification of the remaining specimens provided by Passos et al. (2022) was unsubstantiated, not all of them were unwarranted (see Table 4). We agree that DHMECN 7644 (identified as *A.lehmanni* Boettger, 1898 in Arteaga et al. 2017) and IBSP 71932 (identified as *A.zebrinus* Jan, 1862 in Grazziotin et al. 2012) are misidentified, but their new identifications provided by Passos et al. (2022) are not correct either (see Table 4). DHMECN 7644 is a paratype of *A.michaelsabini* sp. nov., as defined herein, and IBSP 71932 is probably an *A.trihedrurus* Amaral, 1926, not an “*A.triherurus*.” Although the latter probably represents a typo and is a minor error, the problems with the remaining reidentifications are not trivial. For example, Passos et al. (2022) reidentified the same specimen, MZUTI 3758, as *A.iridescens* Peracca, 1896 and also as A.cf.iridescens. Additionally, these authors completely reidentified the type series of both *A.cerberus* and *A.esepe*Arteaga et al., 2017, probably without much confidence since this action is not explained elsewhere in their work and is not trivial. Since MZUTI 4330 and MZUTI 3758 are name-bearing specimens, reidentification of these holotypes as *A.iridescens*, A.cf.iridescens, or anything other than their original identification presented in Arteaga et al. (2017) implies that these species are not valid. Surprisingly, the fact that the taxonomic validity of these two species is not questioned elsewhere in Passos et al. (2022) suggests that some of these reidentifications were proposed carelessly. Thus, in Table 4, we evaluate these reidentifications and mention whether they are substantiated or warranted or neither. Finally, we propose the reidentification of an additional six *Atractus* specimens (Table 5) having sequences deposited in GenBank based on the results presented in Fig. 1.

**Table 4.** Reidentification of *Atractus* specimens reidentified in Passos et al. 2022 based on direct examination of voucher specimens.

Voucher	Original identification (Arteaga et al. 2017)	Proposed reidentification (Passos et al 2022)	Reidentification warranted and substantiated	Identification
MZUTI 4330	*Atractuscerberus*	Atractuscf.iridescens	No	*Atractuscerberus*
MZUTI 1385, 2649–50, 3323	*Atractusoccidentalis*	*Atractusdunni*	No	*Atractusmicrorhynchus*
MZUTI 3758–59	*Atractusesepe*	Atractuscf.iridescens and *A.iridescens*	No	*Atractusesepe*
MZUTI 4178	*Atractusiridescens*	*Atractusiridescens*	Identity remained the same, but listed as “reidentified”	*Atractusiridescens*
MZUTI 4122	*Atractusmicrorhynchus*	*Atractusiridescens*	No	*Atractusmicrorhynchus*
DHMECN 7644	*Atractuslehmanni*	*Atractusroulei*	Warranted at time of publication	*Atractusmichaelsabini* sp. nov.
MZUTI 5109	*Atractusmicrorhynchus*	*Atractusdunni*	No	*Atractusmicrorhynchus*
MZUTI 5107	*Atractuspyroni*	*Atractusroulei*	Yes	*Atractusroulei*
ANF 2390	*Atractustouzeti*	*Atractuspachacamac*	Yes	*Atractuspachacamac*
GFM 307	*Atractusschach*	*Atractussnethlageae*	Yes	*Atractussnethlageae*
IBSP 71932	*Atractuszebrinus*	*Atractustriherurus*	Yes, but name misspelled	*Atractustrihedrurus*

**Table 5.** Reidentification of *Atractus* sequences available in GenBank based on direct examination of voucher specimens.

Voucher	GenBank accession numbers	Identity in GenBank	Identification
DHMECN 8343	KY610059, KY610105	*Atractusmajor*	*Atractusarangoi*
QCAZ 7887	MT507872, MT511989	*Atractusroulei*	*Atractusmichaelsabini* sp. nov.
QCAZ 7889	MT507874, MT511990	*Atractusroulei*	*Atractusmichaelsabini* sp. nov.
QCAZ 9643	MT507875, MT511981, MT511991	*Atractusroulei*	*Atractusmichaelsabini* sp. nov.
QCAZ 9652	MT507876, MT511992	*Atractusroulei*	*Atractusmichaelsabini* sp. nov.
MHUA 14368	GQ334664, GQ334581, GQ334558, GQ334480	*Atractuswagleri*	*Atractuslasallei*

## ﻿Discussion

*Atractus* is perhaps the most taxonomically complex snake genus and the work needed to elucidate its evolutionary relationships is just starting. Achieving a comprehensive understanding of the real diversity within this cryptozoic group of snakes will require an approach combining three actions: 1) improving the taxon sampling available for comparison at the molecular level; 2) re-sampling type localities as well as exploring new remote areas; and 3) defining species boundaries among *Atractus* species using an integrative taxonomic approach, not only scale counts. Below, we discuss how our results help clear the waters in *Atractus* taxonomy and provide insights on where future research efforts might be most effective.

The molecular phylogenies presented here (Fig. 1 and Suppl. material 2: Fig. S1) include only approximately 30% of the total known diversity of the genus *Atractus*; thus, many higher-level relationships within species groups are still unknown. The placement of *A.trilineatus* as sister to a clade containing *A.arangoi* and *A.major*, rather than as an early divergent *Atractus* species (Murphy et al. 2019) is puzzling, but this relationship is moderately supported in both the BI and ML analyses and will likely benefit from an improved sampling of molecular characters. *Atractusarangoi* is supported as a valid species in our molecular analyses and is easily diagnosable from *A.major* based on body size, coloration, and lepidosis (Table 3), confirming its status as a valid species (Prado 1939; Daniel 1949). With the exception of the weakly placed *A.zidoki* Gasc & Rodrigues, 1979, we found that cis-Andean species of *Atractus* are more closely related to other cis-Andean species, whereas trans-Andean ground snakes are more closely related to other trans-Andean species. This finding may prove useful in understanding why the presence of the same *Atractus* species on both sides of the Andes, a scenario suggested for *A.gigas* by Passos et al. (2010), is unlikely.

There is a clade formed by the remaining Ecuadorian *Atractus* that were included in the phylogeny and are distributed along the Amazonian slopes of the Andes. The new species, *A.discovery* sp. nov. and *A.zgap* sp. nov., are included in this group. While the former is the strongly supported sister species to *A.resplendens*, it has a coloration pattern most similar to *A.orcesi* (Fig. 5e), a species not previously included in any phylogenetic analyses and characterized by having a yellow belly with a black ventral stripe. The black stripe on a yellow belly is a characteristic shared by *A.duboisi*, *A.discovery* sp. nov., and *A.orcesi*, but is absent from *A.resplendens* and *A.ecuadorensis* (the other two members of the group) and confirms this as a useful character in diagnosing species within this clade. In the ML analysis (Suppl. material 2: Figure S1), *A.dunni* is nested within *A.microrhynchus*, a topology not recovered in the BI phylogeny or in previous analyses despite being based on the same DNA sequences. We believe this incongruence is the result of character sampling and methodological approach instead of these two species being conspecific. The phylogenetic position of *A.zgap* sp. nov., a snake most similar to *A.ecuadorensis* in size, coloration, and lepidosis, as sister to a clade of banded Amazonian *Atractus* rather than to *A.ecuadorensis* is puzzling. Although the placement of *A.zgap* sp. nov. in both the BI and ML analyses is strongly supported and is probably correct, we do not have as much confidence in the position of *A.ecuadorensis* and this may be explained by the fact that only one gene fragment (ND4) was available for the latter species (Appendix I). We found higher intraspecific topological distances between members of *A.carrioni*, *A.major*, and *A.roulei* than between the pair of species *A.trefauti*-*A.schach*. Therefore, attention should be given to reevaluating the validity of these species.

The binary environmental niche models (Fig. 2) for both *Atractusmichaelsabini* sp. nov. and *A.roulei* include xeric inter-Andean valleys where populations of these snakes are known to occur, even though elsewhere these species inhabit humid areas where the dominant vegetation cover is evergreen montane forest (Sierra 1999). We found that the deep intraspecific genetic divergence found within both of these taxa corresponds to the sampling of populations distributed on different bioclimatic regimes (i.e., snakes of xeric habitats are genetically distinct from snakes of humid habitats). Although we did not find morphological differences that would allow the distinction of these subpopulations, we do not rule out the possibility that they correspond to cryptic species diversity.

In addition to creating a more robust phylogenetic tree of ground snakes, one of the most important actions in the quest towards a more clear, stable, and useful *Atractus* taxonomy is the correct identification of museum specimens. Based on our review of the reidentifications proposed in Passos et al. (2022), it is evident that reassigning the species identities of museum vouchers is not a trivial pursuit. On the contrary, it has consequences that go beyond taxonomy. For example, reidentifying the only known museum specimens of the Critically Endangered *A.cerberus* as *A.iridescens*, a Least Concern species, implies that the population of this species in the isolated Pa­coche forest of west-central Ecuador is not as unique and worthy of conservation efforts. It also implies that the presence of a species endemic to the humid Chocó rainforest in an isolated mountain range belonging to another biogeographic province is likely.

The last point on biogeography deserves elaboration. The use of species distribution models can be used not only to discover and test biogeographical patters but also to test species as hypotheses (Ahmadzadeh et al. 2013; Ortega-Andrade et al. 2015). The elaboration of distribution maps using ecological variables, in addition to the presentation of accurate color photographs of specimens and their corresponding genetic information as a part of an integrative taxonomic approach can greatly benefit *Atractus* taxonomy, a branch of herpetology in which diagnoses have largely been based only on meristics (Savage 1960; Passos et al. 2009c; Passos et al. 2010). Using this framework can help prevent *Atractus* species that are valid taxa and occur in distinct biogeographical provinces to be subsumed under the same name on the basis of overlapping scale counts. An example of this are the snakes *A.gigas* and *A.dunni*, two cloud forest species endemic to the Pacific slopes of the Andes in northwestern Ecuador. These snakes present a biogeographic pattern of distribution shared by other co-occurring reptiles (Avila Pires 2001; Köhler et al. 2004; Arteaga et al. 2013; Torres-Carvajal and Lobos 2014; Arteaga et al. 2016). Given how narrow the climatic requirements of these two *Atractus* species are (Mejía Guerrero 2018; Mantilla Espinoza 2021), their presence on the Amazonian slopes of the Andes, or on the Chocoan lowlands, as suggested by Passos et al. (2010) and Passos et al. (2022), respectively, is unlikely. In this work, we presented evidence that supports the status of *A.gigas* and *A.dunni* as species endemic to the cloud forests of the Pacific slopes of the Andes in northwestern Ecuador.

Finally, although *Atractus* systematics have progressed greatly since Savage published his monograph on the Ecuadorian members of this genus in 1960, many “stones are still left unturned.” The Ecuadorian species *A.clarki* Dunn & Bailey, 1939, *A.collaris* Peracca, 1897, *A.gaigeae* Savage, 1955, and *A.occipitoalbus* have not been included in a phylogenetic work, and their status remains uncertain. Also, an overwhelming majority of *Atractus* diversity, both described and undescribed, is in Colombia (Uetz et al. 2022). Unfortunately, only one or two samples of *Atractus* coming from Colombia have been included in published phylogenetic trees of this genus (Arteaga et al. 2017; Murphy et al. 2019; Melo-Sampaio et al. 2021, Passos et al. 2022). Thus, we suggest that future work on *Atractus* be focused on unveiling the incredible diversity of this genus in Colombia.

## Supplementary Material

XML Treatment for Atractus discovery

XML Treatment for Atractus zgap

XML Treatment for Atractus michaelsabini

## ﻿Appendix I

**Table A1.** GenBank accession numbers for loci and terminals of taxa and outgroups sampled in this study. Novel sequence data produced in this study are marked with an asterisk (*).

Species	Voucher	16S	CYTB	ND4	CMOS	NT3	RAG1
*A.arangoi*	DHMECN 8343	KY610059	–	KY610105	–	–	–
*A.arangoi*	ZSFQ 4947	ON907812*	ON925021*	ON925012*	–	–	–
*A.arangoi*	ZSFQ 4948	ON907811*	ON925020*	ON925011*	–	–	–
*A.atlas*	QCAZ 14946	MH790470	MN887669	MN887691	MN887640	MN887715	MN887745
*A.badius*	MNRJ 26717	MH790476	MK835891	–	MK835864	MK835980	MK835948
*A.boimirim*	MPEG 21233	MH790478	–	–	MK835866	MK835982	MK835951
*A.carrioni*	MZUTI 4195	KY610046	–	KY610094	–	–	–
*A.carrioni*	QCAZ 6446	MT507867	–	MT511983	–	–	–
*A.carrioni*	QCAZ 6533	MT507868	–	MT511984	–	–	–
*A.carrioni*	QCAZ 6534	MT507869	–	MT511985	–	–	–
*A.carrioni*	QCAZ 10038	MT507864	MT511977	MT511982	–	–	–
*A.carrioni*	QCAZ 13094	MT507865	MT511978	–	–	–	–
*A.cerberus*	MZUTI 4330	KY610047	KY610073	KY610095	–	–	–
*A.dapsilis*	MNRJ 16796	MH790480	MK835894	MK835926	MN887642	MN887716	MK835951
*A.discovery* sp. nov.	MZUA.Re.466	OP225330*	OP244686*	OP225393*	–	–	–
*A.duboisi*	MZUTI 62	KT944041	–	KT944059	–	–	–
*A.dunni*	MZUTI 2189	KY610048	–	KY610096	–	–	–
*A.dunni*	MZUTI 3031	KY610049	–	KY610097	–	–	–
*A.dunni*	MZUTI 4318	KY610050	KY610074	KY610098	–	–	–
*A.dunni*	MZUTI 4319	KY610051	KY610075	KY610099	–	–	–
*A.ecuadorensis*	DHMECN 5105	–	–	KY610100	–	–	–
*A.elaps*	QCAZ 5574	MN855378	MK835896	MN887692	MK835867	MN887717	MK835954
*A.esepe*	MZUTI 3758	KY610053	KT944052	KY610102	–	–	–
*A.esepe*	MZUTI 3759	KT944039	KT944051	KT944058	–	–	–
*A.favae*	MZUSP 20211	MN855380	MN887670	–	–	–	–
*A.flammigerus*	MNRJ 26720	MH790488	MK835903	MK835932	MK835873	MK835994	–
*A.gigas*	MZUTI 3286	KT944043	KT944053	MN891764	–	–	–
*A.iridescens*	DHMECN 9633	KY610054	KY610077	–	–	–	–
*A.iridescens*	MZUTI 3548	KY610055	KY610078	–	–	–	–
*A.iridescens*	MZUTI 3680	KY610056	KY610079	–	–	–	–
*A.iridescens*	MZUTI 4178	KT944040	KY610080	–	MH374931	–	–
*A.iridescens*	MZUTI 4697	KY610057	KY610081	–	–	–	–
*A.lasallei*	MHUA 14368	–	GQ334480	GQ334581	–	–	–
*A.latifrons*	MPEG 22630	MH790493	MK835908	MN887694	MK835875	–	–
*A.major*	ANF 1545	KT944045	–	KY610104	–	–	–
*A.major*	CORBIDI 223	MH790497	–	–	–	–	–
*A.major*	MNRJ 26126	MH790498	MK835911	–	–	–	MK835958
*A.major*	MZUSP 20868	MH790499	–	–	–	–	–
*A.major*	MZUSP 20887	MH790500	–	–	–	–	–
*A.major*	QCAZ 4691	MH790506	MK835912	MK835934	MN887643	MK836002	MN887747
*A.major*	QCAZ 4993	MH790507	–	MK835935	–	–	–
*A.major*	QCAZ 5891	MH790508	MK835913	MK835936	MK835878	MK836003	MK835962
*A.major*	QCAZ 7881	MH790509	MK835914	MK835937	–	MK836004	MK835963
*A.major*	QCAZ 13819	MH790504	–	MK835933	–	MK836000	MK835960
*A.major*	UFACRB 532	MH790511	MK835915	–	MK835879	MK836005	–
*A.michaelsabini* sp. nov.	AMARU 002	ON907809*	ON925018*	ON925009*	–	–	–
*A.michaelsabini* sp. nov.	MZUTI 5289	ON907810*	ON925019*	ON925010*	–	–	–
*A.michaelsabini* sp. nov.	DHMECN 7644	KY610058	KY610082	KY610103	–	–	–
*A.michaelsabini* sp. nov.	QCAZ 7887	MT507872	–	MT511989	–	–	–
*A.michaelsabini* sp. nov.	QCAZ 7889	MT507874	–	MT511990	–	–	–
*A.michaelsabini* sp. nov.	QCAZ 9643	MT507875	MT511981	MT511991	–	–	–
*A.michaelsabini* sp. nov.	QCAZ 9652	MT507876	–	MT511992	–	–	–
*A.michaelsabini* sp. nov.	ZSFQ 4939	ON907808*	ON925017*	ON925008*	–	–	–
*A.microrhynchus*	MZUTI 1385	KY610063	KY610086	KY610109	–	–	–
*A.microrhynchus*	MZUTI 2649	KY610064	KY610087	KY610110	–	–	–
*A.microrhynchus*	MZUTI 2650	KT944038	KT944050	KT944057	–	–	–
*A.microrhynchus*	MZUTI 3323	KY610065	KY610088	KY610111	–	–	–
*A.microrhynchus*	MZUTI 4122	KT944037	KT944049	KT944056	–	–	–
*A.microrhynchus*	MZUTI 5109	KY610060	KY610083	KY610106	–	–	–
*A.modestus*	MZUTI 4760	KY610061	KY610084	KY610107	–	–	–
*A.multicinctus*	MZUTI 5106	KY610062	KY610085	KY610108	–	–	–
*A.orcesi*	ZSFQ 2222	ON907807*	–	ON925007*	–	–	–
*A.orcesi*	ZSFQ 2237	ON907806*	ON925016*	ON925006*	–	–	–
*A.pachacamac*	QCAZ 12630	MH790524	MN887672	MN887697	MN887647	MN887723	MN887751
*A.paucidens*	MZUTI 5102	KY610066	ON925015*	KY610112	–	–	–
*A.resplendens*	MZUTI 3996	KT944042	KT944055	KT944060	–	–	–
*A.riveroi*	MNRJ 26087	MH790526	MK835916	–	–	MK836006	MK835964
*A.roulei*	MZUTI 4503	KY610069	KY610090	KY610116	–	–	–
*A.roulei*	MZUTI 4544	KY610069	KY610091	KY610117	–	–	–
*A.roulei*	MZUTI 5107	KY610068	KY610089	KY610115	–	–	–
*A.roulei*	QCAZ 6256	–	MT511980	MT511988	–	–	–
*A.roulei*	QCAZ 7192	MT507871	MT511980	–	–	–	–
*A.roulei*	ZSFQ 4945	ON907805*	ON925014*	ON925005*	–	–	–
*A.savagei*	MZUTI 4916	KY610070	KY610092	KY610118	–	–	–
*A.schach*	AF 1716	MH790527	MK835917	–	MK835880	MK836007	–
*A.snethlageae*	MPEG 20605	MH790513	MN887678	MN887705	MN887655	MN887731	MN887759
*A.tartarus*	MPEG 23931	MH790529	MK835919	MK835938	–	MK836009	MK835965
*A.torquatus*	MPEG 23686	MH790532	MK835921	MK835941	–	MK836012	MK835968
*A.touzeti*	ZSFQ 4949	ON907804*	ON925013*	ON925004*	–	–	–
*A.trefauti*	MNRJ 26709	MH790536	MK835923	MK835942	MK835883	MK836015	MK835971
*A.trilineatus*	CAS 257740	MK648018	MK648027	MK648035	MK648043	–	–
*A.trilineatus*	UWISM 2015.18.2	MK648014	MK648022	MK648031	MK648039	–	–
*A.typhon*	MZUTI 3284	KT944044	KT944054	KT944062	–	–	–
*A.ukupacha*	QCAZ 4944	MH790540	MN887689	MN887714	MN887668	MN887744	MN887774
*A.zgap* sp. nov.	MZUTI 5311	ON907803*	–	ON925003*	–	–	–
*A.zidoki*	MNHN 1997.2046	AF158487	–	–	–	–	–
*G.godmani*	MVZ 233298	JQ598877	JQ598932	–	–	–	–
*S.nebulatus*	MVZ 233298	EU728583	EU728583	EU728583	–	–	–

## ﻿﻿Appendix II

**Table A2.** List of PCR and sequencing primers and their respective PCR conditions (denaturation, annealing, extension, and number of corresponding cycles) used in this study. All PCR protocols included an initial 3-min step at 94 °C and a final extension of 10 min at 72 °C.

Locus	Primer	Sequence (5’-3’)	Reference	PCR profile
16S	16Sar-L	CGCCTGTTTATCAAAAACAT	Palumbi et al. (1991)	30 cycles of 94 °C (45 sec), 53 °C (45 sec), 72 °C (1 min)
	16Sbr-H-R	CCGGTCTGAACTCAGATCACGT		
Cytb	L14910	GACCTGTGATMTGAAAACCAYCGTTGT	Burbrink et al. (2000)	94 °C (1 min), 58 °C (1 min), 72 °C (2 min) [x30–36]
	H16064	CTTTGGTTTACAAGAACAATGCTTTA		
ND4	ND4	CACCTATGACTACCAAAAGCTCATGTAGAAGC	Arévalo et al. (1994)	94 °C (25 sec), 56 or 60 °C (1 min), 72 °C (2 min) [x25–30]
	Leu	CATTACTTTTACTTGGATTTGCACCA		
	S78	CCTTGGGTGTGATTTTCTCACCT		

## Supplementary materials

Supplementary material 1 Table S1 Data type excel file. Explanation note Locality data for species included in Fig. 2. https://binary.pensoft.net/file/744150This dataset is made available under the Open Database License (http://opendatacommons.org/licenses/odbl/1.0/). The Open Database License (ODbL) is a license agreement intended to allow users to freely share, modify, and use this Dataset while maintaining this same freedom for others, provided that the original source and author(s) are credited.Alejandro Arteaga, Amanda Quezada, Jose Vieira, Juan M. Guayasamin

Supplementary material 2 Figure S1 Data type Image. Explanation note Phylogenetic relationships within *Atractus* inferred using a maximum-likelihood approach and derived from analysis of 3,985 bp of DNA (gene fragments 16S, cytb, ND4, c-mos, NT3, and RAG1). Support values on intra-specific branches are not shown for clarity. Voucher numbers for sequences are indicated for each terminal. Black dots indicate clades with bootstrap values from 90–100%. Grey dots indicate values from 70–89%. White dots indicate values from 50–69% (values < 50% not shown). Colored clades correspond to the species’ distribution presented in the map of Fig. 2. New or resurrected species are indicated in bold type. https://binary.pensoft.net/file/744151This dataset is made available under the Open Database License (http://opendatacommons.org/licenses/odbl/1.0/). The Open Database License (ODbL) is a license agreement intended to allow users to freely share, modify, and use this Dataset while maintaining this same freedom for others, provided that the original source and author(s) are credited.Alejandro Arteaga, Amanda Quezada, Jose Vieira, Juan M. Guayasamin
