# Supplementary material for: ﻿Leaving no stone unturned: three additional new species of Atractus ground snakes (Serpentes, Colubridae) from Ecuador discovered using a biogeographical approach
Source: Zookeys. 2022 Sep 15;1121:175–210. doi: 10.3897/zookeys.1121.89539 (PMC9848674; doi:10.3897/zookeys.1121.89539)
Supplement: Supplementary material 2 — Figure S1 [file zookeys-1121-175_article-89539__-s002.pdf]

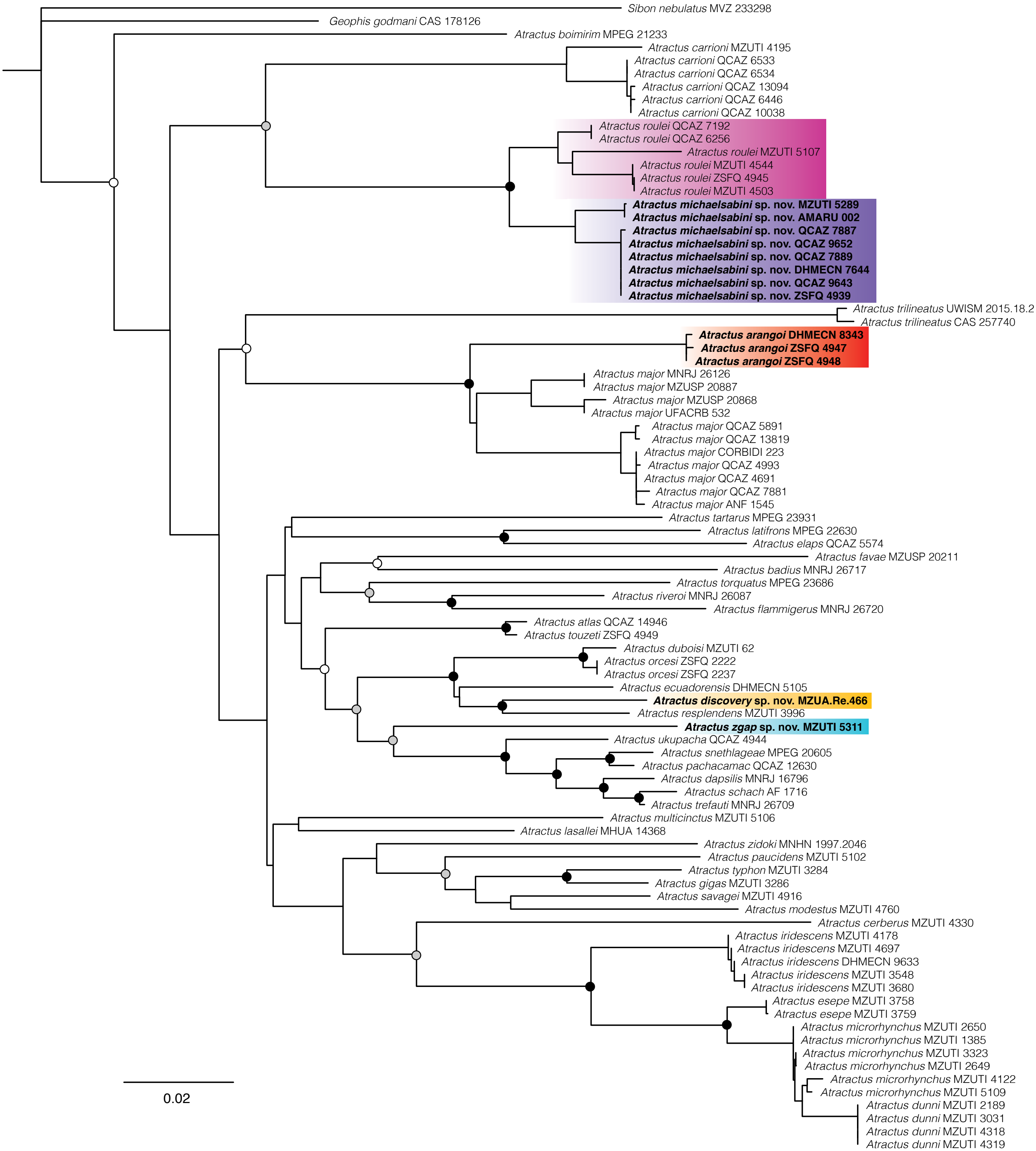
Sibon nebulatus MVZ 233298
Geophis godmani CAS 178126
Atractus boimirim MPEG 21233
Atractus carrioni MZUTI 4195
Atractus carrioni QCAZ 6533
Atractus carrioni QCAZ 6534
Atractus carrioni QCAZ 13094
Atractus carrioni QCAZ 6446
Atractus carrioni QCAZ 10038
Atractus roulei QCAZ 7192
Atractus roulei QCAZ 6256
Atractus roulei MZUTI 5107
Atractus roulei MZUTI 4544
Atractus roulei ZSFQ 4945
Atractus roulei MZUTI 4503
Atractus michaelsabini sp. nov. MZUTI 5289
Atractus michaelsabini sp. nov. AMARU 002
Atractus michaelsabini sp. nov. QCAZ 7887
Atractus michaelsabini sp. nov. QCAZ 9652
Atractus michaelsabini sp. nov. QCAZ 7889
Atractus michaelsabini sp. nov. DHMECN 7644
Atractus michaelsabini sp. nov. QCAZ 9643
Atractus michaelsabini sp. nov. ZSFQ 4939
Atractus trilineatus UWISM 2015.18.2
Atractus trilineatus CAS 257740
Atractus arangoi DHMECN 8343
Atractus arangoi ZSFQ 4947
Atractus arangoi ZSFQ 4948
Atractus major MNRJ 26126
Atractus major MZUSP 20887
Atractus major MZUSP 20868
Atractus major UFACRB 532
Atractus major QCAZ 5891
Atractus major QCAZ 13819
Atractus major CORBIDI 223
Atractus major QCAZ 4993
Atractus major QCAZ 4691
Atractus major QCAZ 7881
Atractus major ANF 1545
Atractus tartarus MPEG 23931
Atractus latifrons MPEG 22630
Atractus elaps QCAZ 5574
Atractus favae MZUSP 20211
Atractus badius MNRJ 26717
Atractus torquatus MPEG 23686
Atractus riveroi MNRJ 26087
Atractus flammigerus MNRJ 26720
Atractus atlas QCAZ 14946
Atractus touzeti ZSFQ 4949
Atractus duboisi MZUTI 62
Atractus orcesi ZSFQ 2222
Atractus orcesi ZSFQ 2237
Atractus ecuadorensis DHMECN 5105
Atractus discovery sp. nov. MZUA.Re.466
Atractus resplendens MZUTI 3996
Atractus zgap sp. nov. MZUTI 5311
Atractus ukupacha QCAZ 4944
Atractus snethlageae MPEG 20605
Atractus pachacamac QCAZ 12630
Atractus dapsilis MNRJ 16796
Atractus schach AF 1716
Atractus trefauti MNRJ 26709
Atractus multicinctus MZUTI 5106
Atractus lasallei MHUA 14368
Atractus zidoki MNHN 1997.2046
Atractus paucidens MZUTI 5102
Atractus typhon MZUTI 3284
Atractus gigas MZUTI 3286
Atractus savagei MZUTI 4916
Atractus modestus MZUTI 4760
Atractus cerberus MZUTI 4330
Atractus iridescens MZUTI 4178
Atractus iridescens MZUTI 4697
Atractus iridescens DHMECN 9633
Atractus iridescens MZUTI 3548
Atractus iridescens MZUTI 3680
Atractus esepe MZUTI 3758
Atractus esepe MZUTI 3759
Atractus microrhynchus MZUTI 2650
Atractus microrhynchus MZUTI 1385
Atractus microrhynchus MZUTI 3323
Atractus microrhynchus MZUTI 2649
Atractus microrhynchus MZUTI 4122
Atractus microrhynchus MZUTI 5109
Atractus dunni MZUTI 2189
Atractus dunni MZUTI 3031
Atractus dunni MZUTI 4318
Atractus dunni MZUTI 4319
0.02
